# Does a bicycle accident as the cause of proximal femur fracture indicate that geriatric co-management is superfluous? A retrospective cohort study

**DOI:** 10.1186/s11556-023-00315-6

**Published:** 2023-03-02

**Authors:** Petra E. Spies, Malene Fix, Benjamin L. Emmink, Tjard R. Schermer

**Affiliations:** 1 Department of Geriatric Medicine and Centre of Excellence for Old Age Medicine, Gelre Hospitals, Apeldoorn & Zutphen, the Netherlands; 2 Department of Surgery, Gelre Hospitals, Apeldoorn & Zutphen, the Netherlands; 3 Department of Primary and Community Care, Radboud Institute for Health Sciences, Radboud University Medical Center, PO Box 9101, 6500HB Nijmegen, The Netherlands; 4 Science Support Office, Gelre Hospitals, Apeldoorn & Zutphen, the Netherlands

**Keywords:** Geriatric traumatology, Bicycle, E-bike, Femur fracture, Co-management, Length of stay, Complications

## Abstract

**Background:**

Deployment of geriatric care would be more sustainable if we could limit geriatric co-management to older hip fracture patients who benefit most from it. We assumed that riding a bicycle is a proxy of good health and hypothesized that older patients with a hip fracture due to a bicycle accident have a more favorable prognosis than patients whose hip fracture was caused by another type of accident.

**Methods:**

Retrospective cohort study of hip fracture patients ≥ 70 years admitted to hospital. Nursing home residents were excluded. Primary outcome was length of hospital stay (LOS). Secondary outcomes were delirium, infection, blood transfusion, intensive care unit stay and death during hospitalization. The group with a bicycle accident (BA) was compared to the non-bicycle accident (NBA) group using linear and logistic regression  models, with correction for age and sex.

**Results:**

Of the 875 patients included, 102 (11.7%) had a bicycle accident. BA patients were younger (79.8 versus 83.9 years, *p* < 0.001), less often female (54.9 versus 71.2%, *p* = 0.001) and lived independently more often (100 versus 85.1%, *p* < 0.001). Median LOS in the BA group was 0.91 times the median LOS in the NBA group (*p* = 0.125). For none of the secondary outcomes the odds ratio favored the BA group, except for infection during hospital stay (OR = 0.53, 95%CI 0.28–0.99; *p* = 0.048).

**Conclusions:**

Although older hip fracture patients who had a bicycle accident appeared more healthy than other older hip fracture patients, their clinical course was not more favorable. Based on this study, a bicycle accident is not an indicator that geriatric co-management can be omitted.

## Background

Proximal femur fractures (hip fractures) have serious impact on older people, as morbidity and mortality rates are above 30% in the first year after the fracture [1–4]. A mere half of the patients have regained their pre-accident functional status a year after surgery and one in every four patients who were ADL-independent before their hip fracture ends up in a nursing home [1, 2, 5].

The risk of complications related to the hip surgery is affected by pre-accident functional status as well as by age, sex, body mass index, length of hospital stay, and the time elapsed between the accident and surgery [1, 4, 6]. Early active walking and physical exercise reduce the complication rate as well as the need for prolonged inpatient treatment [4].

Geriatric co-management of older hip fracture patients has been shown to lead to shorter hospital stay, fewer complications, reduced readmission rates, lower costs, better health-related quality of life, and lower mortality. [1, 4, 5] Also, with geriatric co-management of older hip fracture patients the hospital discharge location is more often a rehabilitation center rather than a nursing home [5].

Based on these results, the Dutch guideline for the management of hip fractures in older patients recommends to involve a geriatrician in the management of every admitted older (i.e. ≥ 70 years) hip fracture patient and this is common practice in most hospitals in the Netherlands [7, 8]. However, the growing older population, their increasing pressure on the healthcare system and the shortage of healthcare professionals call for a more efficient approach [9]. Limiting geriatric co-management to older hip fracture patients that are likely to benefit most from it would make their in-hospital management more (cost-)efficient and sustainable for the future.

Clearly the criteria for such a selection process should be based on evidence-based prognostic factors and easy to implement in clinical practice. One potential prognostic factor that—at least to our knowledge—has not been investigated yet is whether or not the hip fracture was caused by a bicycle accident. Previous research has shown that outdoor falls have a lower complication rate compared to indoor falls, the former also being associated with younger age and better health status at the time of injury [3, 10]. As cycling is associated with a good health status [11] and, consequently, riding a bike may be an easy to identify proxy of good health, this may be a useful and easy to apply criterion when selecting older hip fracture patients for omitting geriatric co-management.

In this study we hypothesized that older (≥ 70 years) patients who are admitted to our hospital with a hip fracture due to a bicycle accident have a more favorable prognosis in terms of length of hospital stay, risk of complications during hospitalization and discharge destination compared to older subjects whose hip fracture was caused by another type of accident.

## Methods

### Design and study population

For this retrospective cohort study we collected data from electronic medical records in Gelre Hospitals, an ~400-bed teaching hospital with two locations in the cities of Apeldoorn and Zutphen, both situated in the eastern part of the Netherlands.

In order to be included in the study a patient had to be aged 70 years or older; have a hip fracture; and be admitted to our hospital between January 2018 and December 2020. These patients were all entered in the hospital’s standardized treatment pathway for hip fractures, including routine geriatric co-management. We excluded patients who lived in nursing homes, because nursing home residents are highly unlikely to ride a bicycle and are generally too frail to be exempted from geriatric co-management.

### Data collection and outcomes

Due to its retrospective nature and use of (deidentified) data that is routinely recorded in patients’ medical records the study does not require informed consent by patients nor approval by a medical ethics review committee [12]. Gelre hospitals’ institutional review board formally declared that the study was considered to be exempted from ethics review (file number: 2020–046). The data were retrieved from the electronic patient records and entered in a study database using the cloud-based clinical data management platform Castor EDC (https://www.castoredc.com). For each patient one of the authors (MF) or the department’s research nurse (MvN) assessed from the information in the medical record whether or not the hip fracture had been caused by a bicycle accident or not. In case of doubt with regard to the cause of the fracture the principal investigator (PS) assessed the patient record concerned. The primary outcome for the study was length of hospital stay (in days). Secondary outcomes were: change in living situation after hospital discharge; delirium during hospital stay (yes/no); any infection(s) during hospital stay (yes/no); blood transfusion(s) during hospital stay (yes/no); intensive care unit stay during hospital stay (yes/no); and death during hospitalization (yes/no). To define ‘change in living situation’ we compared each patient’s living situation before the hip fracture occurred (i.e., at home without ADL support; at home with ADL support; at a residential care center; or other (i.e., transfer from a different hospital; hospice; or foreign country) and, based on the discharge destination, assessed whether or not this had regressed to a living situation in which the patient was more dependent on support from others. Type of fracture and type of treatment were recorded for all patients. For the bicycle accident patients we also collected information on the type of bicycle (E-bike; regular bike; or unknown). If patients had experienced more than one hip fracture in the study period of three years, we used data of the most recent one.

### Statistical analysis

For the analyses the study sample was split into a bicycle accident (BA) group and a non-bicycle accident (NBA) group. Normally distributed variables were expressed as means and standard deviations (SD), variables with skewed distributions as medians and interquartile ranges (IQR). Univariate analyses of baseline characteristics, primary and secondary outcomes were performed with Mann–Whitney U test, Student-t test, Pearson Chi-square test or Fisher’s exact test, as appropriate. Because of its skewed distribution the primary outcome variable ‘length of hospital stay’ was converted into a normal distribution using natural log (Ln) transformation before further analysis.

Linear regression analysis was used to test for a difference in log transformed length of hospital stay between the BA and NBA groups. The outcome was back transformed to obtain a ratio of the median number of admission days in the BA and NBA groups. Next, the study sample was divided based on days until discharge: within 8 days, or 8 days or later after admission. The tipping point at 8 days was chosen because it is the average length of hospital stay for hip fracture patients as reported in the Statistics Netherlands’ database [13]. Logistic regression was performed to estimate the odds of being discharged 8 days or later for the BA compared to the NBA group. Patients who died within 8 days of hospital admission were excluded from this analysis.

All secondary outcomes (i.e., change in living situation after hospital discharge; delirium during hospital stay; infection(s) during hospital stay; blood transfusion(s) during hospital stay; intensive care unit stay during hospital stay; death during hospitalization) were analyzed with logistic regression.

Analyses were first performed crude and then with correction for age and sex. Because we considered riding a bicycle a proxy of good health, we deliberately chose not to correct for other factors that are associated with good health, such as (the absence of) dementia or the need for ADL support.

Statistical analyses were performed with SPSS version 25.0. *P* < 0.05 was considered the threshold for statistical significance.

## Results

### Study population

During the 3-year study period a total of 1,031 patients ≥ 70 years were admitted to the hospital with a hip fracture. Nursing home residents (*n* = 156; 15.1%) were excluded. The final study sample (*n* = 875) consisted of 102 patients (11.7%) in which a bicycle accident was the cause of the hip fracture (BA group) and 773 (88.3%) in which it was not (NBA group). In 83.3% of the BA group the type of bicycle the patient was riding at the time of the accident was not recorded in the medical record. For those in which it was recorded (*n* = 20) the majority (*n* = 16, 80%) had been riding an E-bike, the others (*n* = 4, 20%) a regular bike.

Table 1 shows the baseline characteristics of the total study sample and the BA and NBA subgroups. The percentage of females in the BA group was 16% lower compared to the NBA group (54.9 *versus* 71.2%, *p* = 0.001) and the BA patients were, on average, 4.1 years younger than the NBA patients (79.8 (SD 5.8) *versus* 83.9 (SD 6.9) years, *p* < 0.001). The majority of BA patients lived at home without ADL support (96.1 *versus* 58.1% in NBA patients, *p* < 0.001).

**Table 1:** Baseline characteristics of 875 patients admitted to hospital with a hip fracture in the period 2018 to 2020

	*Total study sample*	*Cause of hip fracture*		
	(*n* = 875)	*Bicycle accident (BA)* (*n* = 102)	*Non-bicycle accident (NBA)* (*n* = 773)	*p*-value^§^
Females	606 (69.3)	56 (54.9)	550 (71.2)	0.001^$^
Age in years, mean (SD)	83.4 (6.9)	79.8 (5.8)	83.9 (6.9)	< 0.001^*^
Dementia	149 (17.0)	4 (4.0)	145 (18.7)	< 0.001^&^
Living situation before hip fracture				< 0.001^&^
at home without ADL support	547 (62.5)	98 (96.1)	449 (58.1)	
at home with ADL support	213 (24.3)	4 (3.9)	209 (27.0)	
residential care center	106 (12.1)	0 (0)	106 (13.7)	
Other	9 (1.0)	0 (0)	9 (1.0)	
Type of fracture				0.787^$^
medial collum fracture	450 (51.4)	55 (53.9)	395 (51.1)	
pertrochanteric fracture	309 (35.3)	33 (32.4)	276 (35.7)	
subtrochanteric	47 (5.4)	7 (6.9)	40 (5.2)	
periprosthetic fracture	69 (7.9)	7 (6.9)	62 (8.0)	
Type of treatment				< 0.001^&^
hip hemiarthroplasty	344 (39.3)	31 (30.4)	313 (40.5)	
gamma nail	336 (38.4)	34 (33.3)	302 (39.1)	
total hip arthroplasty	44 (5.0)	12 (11.8)	32 (4.1)	
dynamic hip screw	26 (3.0)	8 (7.8)	18 (2.3)	
cannulated hip screws	25 (2.9)	6 (5.9)	19 (2.5)	
other surgical techniques	56 (6.4)	7 (6.9)	49 (6.3)	
non-surgical treatment	44 (5.0)	4 (3.9)	40 (5.2)	

Figures are numbers (%) unless stated otherwise

*ADL* Activities of daily living

^§^ for difference between the BA and NBA subgroups

^*^ from Student-t test

^$^ from Pearson Chi-Square test

^&^ from Fisher’s exact test

### Difference in outcomes

Table 2 shows the univariate analysis of primary and secondary outcomes. Median length of hospital stay was 8.0 in de BA group and 9.0 in the NBA group (*p* = 0.010). NBA patients were more often discharged to a geriatric rehabilitation center or nursing home. BA patients had fewer infections (11.8 *versus* 21.3%, *p* = 0.024). There were no statistically significant differences in ICU stay, delirium, blood transfusions or in-hospital mortality.

**Table 2:** Results of the univariate analysis of primary and secondary outcomes in the bicycle accident and non-bicycle accident groups

	*Cause of hip fracture*		
	*Bicycle accident (BA)* (*n* = 102)	*Non-bicycle accident (NBA)* (*n* = 773)	*p*-value
*Primary outcomes*			
Length of hospital stay in days, median (IQR)	8.0 (5.3)	9.0 (7.0)	0.010^*^
*Secondary outcomes*			
Discharge destination			< 0.001^*&*^
home without ADL support	28 (27.5)	57 (7.4)	
home with ADL support	27 (26.5)	133 (17.2)	
residential care center	0	69 (8.9)	
geriatric rehabilitation center	44 (43.1)	404 (52.3)	
nursing home	1 (1)	50 (6.5)	
other	2 (2)	60 (7.8)	
ICU stay	2 (2)	27 (3.5)	0.565^*&*^
Delirium	15 (14.7)	150 (19.4)	0.254^*$*^
Infection(s)	12 (11.8)	165 (21.3)	0.024^*$*^
Blood transfusion(s)	12 (11.8)	151 (19.5)	0.058^*$*^
Death	1 (1)	26 (3.4)	0.354^*&*^

Figures are numbers (%) unless stated otherwise

^*^ from Mann–Whitney U test

^$^ from Pearson Chi-Square test

^&^ from Fisher’s exact test

Table 3 shows that the back transformed difference in hospital admission days between the BA and NBA groups was 0.88 (95%CI 0.78 to 0.99; *p* = 0.030). The interpretation of this number is that the ratio of the median number of admission days in the BA and NBA group was 0.88 or, in other words, the median number of admission days in the BA group was 0.88 times the median number in the NBA group. When age and sex were entered in the linear regression model the effect was no longer statistically significant (0.91, 95%CI 0.81 to 1.03; *p* = 0.125).

**Table 3:** Results of linear and logistic regression analyses to compare the primary outcome between the bicycle accident (*n* = 102) and non-bicycle accident (*n* = 773) groups

	*Estimate*	*95%CI*	*p value*
**Length of hospital stay**			
*From linear regression analysis, number of admission days*			
Model without covariates:			
Fracture caused by bicycle accident	0.88^a^	0.78, 0.99	0.030
Model with covariates:			
Fracture caused by bicycle accident	0.91^a^	0.81, 1.03	0.125
Age (reference: one year younger)	1.13^a^	1.01, 1.02	< 0.001
Females (reference: males)	0.92^a^	0.85, 1.00	0.056
**Hospital discharge before 8 days**			
*From logistic regression analysis, OR*^b^			
Model without covariates:			
Fracture caused by bicycle accident	0.64	0.42, 0.97	0.034
Model with covariates:			
Fracture caused by bicycle accident	0.73	0.47, 1.12	0.148
Age (reference: one year younger)	1.05	1.02, 1.07	< 0.001
Females (reference: males)	0.78	0.58, 1.06	0.115

Models are with and without age and sex included as covariates

*BA* Bicycle accident, *CI* Confidence interval, *NBA* Non-bicycle accident, *OR* Odds ratio

^a^ transformed back from ln transformed results

^b^ NBA as reference

The odds ratio of being discharged 8 days or later after the admission for the hip fracture was 0.64 (95%CI 0.42 to 0.97; *p* = 0.034) for the BA compared to the NBA group (Table 3). Again, after adding age and sex to the model this effect was no longer statistically significant (OR = 0.73, 95%CI 0.47 to 1.12; *p* = 0.148).

Table 4 shows the results of the logistic regression models for the secondary outcomes. Only for the occurrence of infection during the hospital stay a statistically significant odds ratio in favor of the BA group was seen (OR = 0.49, 95%CI 0.26 to 0.92;*p* = 0.026) which remained statistically significant after adding age and sex to the model (OR = 0.53, 95%CI 0.28 to 0.99; *p* = 0.048). For none of the other secondary outcomes the odds ratio indicated an odds favoring the BA group. Age and/or sex were independently associated with all of the secondary outcomes (see Table 4).

**Table 4:** Results of logistic regression analyses to compare secondary outcomes between the bicycle accident (*n* = 102) and non-bicycle accident (*n* = 773) groups

	*Odds ratio*^a^	*95%CI*	*p value*
**More dependent at discharge destination**			
Model without covariates:			
Fracture caused by bicycle accident	0.66	0.41, 1.06	0.085
Model with covariates:			
Fracture caused by bicycle accident	0.79	0.49, 1.29	0.352
Age (reference: one year older)	1.06	1.03, 1.09	< 0.001
Females (reference: males)	0.83	0.57, 1.22	0.349
**ICU stay**			
Model without covariates:			
Fracture caused by bicycle accident	0.55	0.13, 2.36	0.423
Model with covariates:			
Fracture caused by bicycle accident	0.43	0.10, 1.85	0.256
Age (reference: one year older)	0.98	0.93, 1.03	0.466
Females (reference: males)	0.33	0.16, 0.71	0.004
**Delirium**			
Model without covariates:			
Fracture caused by bicycle accident	0.72	0.40, 1.27	0.256
Model with covariates:			
Fracture caused by bicycle accident	0.74	0.41, 1.34	0.317
Age (reference: one year older)	1.04	1.01, 1.06	0.006
Females (reference: males)	0.54	0.38, 0.77	0.001
**Blood transfusion(s)**			
Model without covariates:			
Fracture caused by bicycle accident	0.55	0.29, 1.03	0.061
Model with covariates:			
Fracture caused by bicycle accident	0.60	0.32, 1.14	0.116
Age (reference: one year older)	1.03	1.00, 1.06	0.026
Females (reference: males)	0.83	0.58, 1.20	0.322
**Infection(s)**			
Model without covariates:			
Fracture caused by bicycle accident	0.49	0.26, 0.92	0.026
Model with covariates:			
Fracture caused by bicycle accident	0.53	0.28, 0.99	0.048
Age (reference: one year older)	1.03	1.00, 1.05	0.022
Females (reference: males)	0.76	0.53, 1.08	0.130
**Death**			
Model without covariates:			
Fracture caused by bicycle accident	0.28	0.04, 2.12	0.220
Model with covariates:			
Fracture caused by bicycle accident	0.23	0.03, 1.77	0.159
Age (reference: one year older)	1.02	0.96, 1.08	0.510
Females (reference: males)	0.23	0.10, 0.61	< 0.001

Models are with and without age and sex included as covariates

*ICU* Intensive care unit, *NBA* Non-bicycle accident

^a^ NBA group as reference group

## Discussion

We assumed that riding a bike is a proxy of good health and hypothesized that older patients with a hip fracture due to a bicycle accident have a more favorable prognosis than patients whose hip fracture was caused by another type of incident. If this were the case, than geriatric co-management could be omitted for patients with a hip fracture due to a bicycle accident, thereby facilitating efficient geriatric care in a time of limited resources and an aging population.

The BA group appeared to have a better health status than the NBA group: they were younger, all lived on their own and most without help, and only a few had dementia. However, this did not translate to a more favorable prognosis. The one day difference in median hospital LOS was explained by age and not by the bicycle accident. The odds of being discharged to a living situation in which the patient was more dependent on support from others was not lower in the BA group and, again, driven by age. Although the odds ratios of complications during hospital stay all seemed to be in favor of the BA group, this was not statistically significant after correction for age and sex, except for the occurrence of (any type of) infection.

Based on these results, riding a bike is not a prognostic factor that can be used to exclude patients from geriatric co-management. It is conceivable that a hip fracture in older patients is such an assault on their health that even being in good condition pre-accident is no guarantee for a favorable prognosis. To illustrate: the younger and more independent BA group in this study still had a delirium incidence of nearly 15%. It is also possible that bicycle accidents, especially with E-bikes, result in higher energy traumas with additional injuries that negatively influence prognosis [14, 15]. Moreover, E-bikes may allow older persons with weak or moderate health and fitness to still ride a bike. This potentially weakens our assumption that older persons who ride a bike are in relatively good shape, which may have reduced the presumed difference in health/fitness between the BA and NBA groups. We have no information about the circumstances of the accidents that led to the hip fractures in this study, and it was largely unknown whether the patients who rode a bicycle were using an E-bike or a regular bike. This is a limitation of our retrospective study. Another limitation is the dichotomous registration of in-hospital complications (present or absent), without information about their severity or duration. We cannot rule out the possibility that complications in the BA group were milder, which would support a more favorable prognosis despite our current findings. However, this has not resulted in a difference in our primary outcome, hospital LOS. With regard to the secondary outcome ‘change in patients’ living situation’ we could not be sure whether a patient’s discharge destination had been just temporarily or was permanent. Final limitations to mention are the fact that we did not collect data of previous proximal femur fractures from the patients’ electronic medical records (and therefore could not correct for this in the statistical analyses) and were limited in collecting data regarding other potentially relevant prognostic factors, as the electronic medical records were the only data source we used. A prospective study would be required to overcome this.

A strength of our study is that it closely represents clinical practice. We have studied a large cohort of older hip fracture patients in a general hospital, where orthogeriatric care is organized in accord with current guidelines [6]. Our results can therefore be generalized to other hospitals providing orthogeriatric care, although we do realize that the proportion of cycling older patients may be different in other (less flat) countries.

## Conclusions

Our study shows that a bicycle accident as the cause of a hip fracture in an older patient is not an unambiguous indication that geriatric co-management can be omitted. It may well be possible that prognostic factors that are easy to identify yet specific enough to not miss frail patients do not exist for the heterogeneous older hip fracture population. A better approach may be to continue geriatric co-management for all hip fracture patients over 70 years old, but to limit the care to the actual individual needs in order to improve its efficiency.

## Data Availability

The datasets used and analyzed during the current study are available from the corresponding author on reasonable request.

## Abbreviations

ADL: Activities of daily living
BA: Bicycle accident
EDC: Electronic data capture
ICU: Intensive care unit
IQR: Interquartile range
Ln: Natural log
LOS: Length of hospital stay
NBA: Non-bicycle accident
OR: Odds ratio
SD: Standard deviation
SPSS: Statistical Package for the Social Sciences
